# Gender differences in pancreatic neuroendocrine neoplasms: A retrospective study based on the population of Hubei Province, China

**DOI:** 10.3389/fendo.2022.885895

**Published:** 2022-08-08

**Authors:** Mengfei Fu, Li Yu, Liu Yang, Yang Chen, Xiao Chen, Qinyu Hu, Hui Sun

**Affiliations:** ^1^ Department of Endocrinology, Union Hospital, Tongji Medical College, Huazhong University of Science and Technology, Wuhan, China; ^2^ Department of Endocrinology, Hubei Provincial Clinical Research Center for Diabetes and Metabolic Disorders, Wuhan, China; ^3^ Department of Emergency Medicine, Union Hospital, Tongji Medical College, Huazhong University of Science and Technology, Wuhan, China

**Keywords:** pancreatic neuroendocrine neoplasms, gender differences, age, functional status, distant metastasis

## Abstract

**Objective:**

The aims of the present study were to investigate gender differences in the clinicopathological features, distant metastasis and prognosis of pancreatic neuroendocrine neoplasms (pNENs) in a Chinese population, and to identify any important gaps in the classification and management of pNENs relative to gender.

**Methods:**

Retrospective collection of the clinicopathological data of 193 patients with pathologically confirmed pNENs were analyzed and follow up was extended to observe the prognosis of the disease. Differences between genders in basic characteristics, clinical symptoms, comorbidities, and tumor parameters were analyzed.

**Results:**

There was no significant difference in females and males, however, moderately higher for females (52.8% vs. 47.2%), with the largest subgroup being 40~60 years of age (54.9%). Age at onset (P=0.002) and age at diagnosis (P=0.005) were both younger in females compared to males. Males lived more in urban areas and females lived more in rural areas (P=0.047). The proportion of smokers and alcohol drinkers was significantly higher in males than in females (P < 0.001). Non-functional pNENs were more frequent in males and functional pNENs in females (P=0.032). In women, functional status of the tumor was significantly associated with metastatic outcome (P=0.007) and functional tumors proved to be a protective factor compared to non-functional tumors (OR=0.090,95% CI: 0.011~ 0.752). There were no gender differences in tumor size, location, grade, stage or prognosis.

**Conclusions:**

Gender differences in some clinicopathological features, and distant metastasis in patients with pNENs were identified, which suggested certain management details that justified emphasis based on gender.

## 1 Introduction

There are gender differences in the occurrence of tumors. Of the 35 major anatomical sites in the human body such as liver, lung and stomach, men have a higher risk of developing tumors than women at 32 of these sites, and even after excluding genital tumors and adjusting for regional and environmental influences, men still dominate in the occurrence of tumors (1). It appears, therefore, that gender might have an independently important impact on tumorigenesis. Furthermore, gender might also influence the spread of tumors (2). The study of tumor development based on gender is an area that still needs to be investigated in depth (3, 4).

Neuroendocrine neoplasms (NENs) originate from neuroendocrine cells and can express neuroendocrine markers. A study by Man D et al. found gender differences in overall survival of the disease, with women having a higher mortality rate than men (5). NENs can occur in the gastrointestinal tract, pancreas, lung, and thymus, with the pancreas being the most common site of occurrence. The National Cancer Institute Surveillance, Epidemiology, and End Results (SEER) reported that the incidence of pNENs has been increasing annually, from 0.18 per 100,000 in 1973 to 0.81 per 100,000 in 2012, which is likely due at least in part to improved detection and increased awareness of the disease among clinicians, but even so, they account for only 1% to 2% of pancreatic tumors (6, 7). Currently, there is a lack of epidemiological data on this disease in China. Reported gender differences in the prevalence of pNENs are inconsistent, and may be related to factors such as race, environment, and lifestyle (5, 8). However, in general, the prevalence is higher in men than in women (9). Moreover, there are reports that gender differences also impact survival time, mortality, and disease prognosis (10, 11). Most published studies on pNENs in China and worldwide have only studied “gender” as a possible risk factor affecting disease prognosis, and rarely analyzed whether there were differences in clinicopathological characteristics between genders.

Therefore, the present study was conducted to determine whether there were gender differences in the clinicopathological features, distant metastasis, and prognosis of pNENs in the Chinese population, and if there were, provide a basis for subsequent gender-based classification and management of pNENs.

## 2 Patients and methods

### 2.1 Study population

This was a retrospective hospital-based study that included patients with pathologically confirmed pNENs from January 2010 to November 2021 at Union Hospital, Tongji Medical College, Huazhong University of Science and Technology, Wuhan, China. Exclusion criteria included the following: patients with incomplete medical records, exocrine pancreatic malignancy, and hereditary diseases such as multiple endocrine neoplasia type 1 (MEN1), neurofibromatosis type 1 (NF1), Von Hippel-Lindau (VHL) syndrome, and tuberous sclerosis complex (TSC). Patient demographics, residence, educational level, clinical symptoms and comorbidities. Additionally, tumor characteristics were recorded including type, grade, stage, location, and size.

The place where the patient lived continuously for more than ten years was defined as the residence, which was divided into urban and rural areas. According to the educational level, patients were divided into the following groups: illiterate, primary school, junior high school, high school and technical secondary school, and college and above. Additional potential risk factors included personal history of smoking (10 cigarettes/day) or drinking (100 ml/day) for ≥3 years prior to admission, and were classified into the smoking or drinking groups.

According to Chinese body mass index (BMI) standards, patients were divided into four subgroups: lean (BMI ≤ 18.4 kg/m^2^), normal (BMI 18.5~23.9 kg/m^2^), overweight (BMI 24.0~27.9 kg/m^2^) and obese (BMI ≥ 28 kg/m^2^).

The location of the tumor was divided into the head or neck and the body or tail of pancreas. Patients were classified according to whether the tumor secreted peptide hormones or biogenic amines and produced corresponding clinical symptoms—functional pancreatic neuroendocrine neoplasms (F-pNENs) and non-functional pancreatic neuroendocrine neoplasms (NF-pNENs), respectively (12). Insulinomas were the most common F-pNENs, and clinical symptoms were caused by hypoglycemia, including autonomic symptoms, neuropsychiatric symptoms, and rarely, symptoms of local tumor compression.

Using the grading criteria in the 2019 World Health Organization (WHO) classification of endocrine organ tumors (13), the tumors were divided into two categories, highly and poorly differentiated. The highly differentiated tumors were further classified according to mitotic rate (2 mm^2^) and Ki-67 index (%): G1 (low grade, mitotic rate: <2 + Ki-67: <3%), G2 (intermediate grade, mitotic rate: 2~20 + Ki-67: 3~20%), and G3 (high grade, mitotic rate: >20 + Ki-67: >20%). Using the 8th American Joint Commission on Cancer (AJCC) TNM staging criteria for pNENs (14), the tumors were classified as stage I (T_1_N_0_M_0_), II (T_2~3_N_0_M_0_), III (T_4_N_0_M_0_, T_any_N_1_M_0_), and IV (T_any_N_any_M_1_). Distant metastases were further subdivided into 3 subtypes: M1a indicated tumor metastasis confined to the liver; M1b indicated tumor metastasis outside the liver, such as lung, ovary, non-regional lymph nodes, peritoneum, and bone; and M1c indicated tumor metastasis in both liver and extra-hepatic organs. In addition, for patients with distant metastases, we analyzed whether there were gender differences and further explored the effect of tumor functional status on metastatic outcomes in different genders.

Follow-up was obtained from the medical record and telephone interviews, and the endpoint event was defined as any form of tumor progression, such as recurrence or metastasis, with a deadline of March 2022. Progression-free survival (PFS) was defined as the time from the day of the patient’s surgery to the occurrence of an endpoint event.

### 2.2 Statistical analysis

The data were statistically analyzed using SPSS 26.0 software. Kolmogorov-Smirnov was used to test whether the continuous variable data conformed to the normal distribution. The data conforming to the normal distribution were expressed as the mean ± standard deviation (SD), otherwise, the median M (P_25_, P_75_) was used. Comparisons between groups were performed using independent samples t-test or nonparametric rank-sum test. Categorical data included two categories of count data and rank data, both expressed as numbers and percentages (n, %), and the chi-square test and nonparametric rank sum test were used for comparison between groups, respectively. P < 0.05 was considered statistically significant.

## 3 Results

### 3.1 Characteristics of patients

#### 3.1.1 Basic characteristics

**Figure 1:**
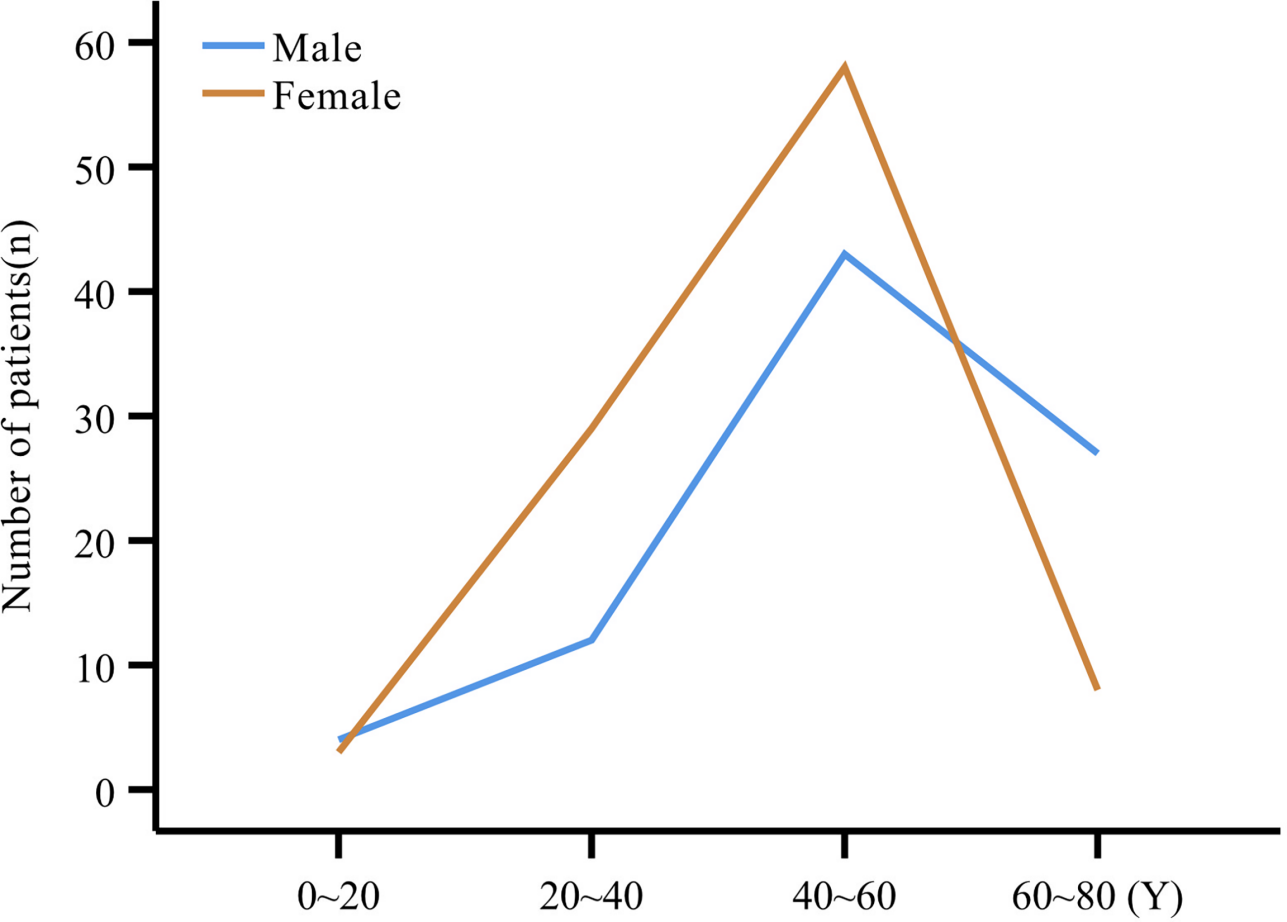
Number of males and females in each age subgroup.

The study comprised 193 patients including 102 females (52.8%) and 91 males (47.2%), with a mean age at onset of 48.4 ± 13.9 years (range:12~80 years), and at diagnosis of 51.0 ± 13.2 years). Patients were grouped into 20-year age intervals, with the highest number of patients (101, 54.9%) in the 40~60 years age subgroup (**Figure 1**). Both the mean age at onset (51.7 ± 14.9 years vs. 45.4 ± 12.4 years, P=0.002) and mean age at diagnosis (53.8 ± 14.1 years vs. 48.5 ± 12.0 years, P=0.005) were higher in men compared to women (**Table 1**).

**Table 1:** Comparison of basic characteristics between different genders.

Parameter	All (N=193)	Male (N=91)	Female (N=102)	Detection value	P value
Age at onset, years	48.4 ± 13.9	51.7 ± 14.9	45.4 ± 12.4	t=3.143	**0.002**
Age at diagnosis, years	51.0 ± 13.2	53.8 ± 14.1	48.5 ± 12.0	t=2.840	**0.005**
BMI, kg/m^2^	23.8 ± 3.7	23.7 ± 3.1	23.9 ± 4.2	t=-0.300	0.765
BMI subgroups, n (%)	177	81	96	z=-0.0067	0.946
≤18.4	12 (6.8%)	5 (6.2%)	7 (7.3%)		
18.5~23.9	90 (50.8%)	38 (46.9%)	52 (54.2%)		
24.0~27.9	45 (25.4%)	31 (38.3%)	14 (14.6%)		
≥28	30 (17.0%)	7 (8.6%)	23 (23.9%)		
Residence, n (%)	182	83	99	x^2^ = 3.956	**0.047**
Urban areas	95 (52.2%)	50 (60.2%)	45 (45.5%)		
Rural areas	87 (47.8%)	33 (39.8%)	54 (54.5%)		
Educational level, n (%)	181	84	97	z=-3.116	**0.002**
Illiterate	15 (8.3%)	1 (1.2%)	14 (14.4%)		
Primary school	18 (9.9%)	5 (6.0%)	13 (13.4%)		
Junior high school	57 (31.5%)	29 (34.5%)	28 (28.9%)		
High school and technical secondary school	36 (19. 9%)	17 (20.2%)	19 (19.6%)		
College and above	55 (30.4%)	32 (38.1%)	23 (23.7%)		
Smoking, n (%)	20 (10.9%)	19 (22. 6%)	1 (1.0%)	x^2^ = 21.798	**<0.001**
Drinking, n (%)	13 (7.1%)	12 (14.3%)	1 (1.0%)	x^2^ = 12.136	**<0.001**

The meaning of the bold values is P<0.05.

The mean BMI of all patients was 23.8 ± 3.7 kg/m^2^, with no statistically significant difference between men and women (P=0.765). After grouping according to BMI, both men (n=38, 46.9%) and women (n=52, 54.2%) were predominantly within the normal range, and there was no gender difference between the subgroups (P=0.946).

#### 3.1.2 Residence

There were modestly more patients from urban than from rural areas (52.2% vs. 47.8%). Statistically, males resided significantly more in urban areas (n=50, 60.2%), and females resided more in rural areas (n=54, 54.5%) (P=0.047). Males were more educated than females (P=0.002) (**Table 1**).

#### 3.1.3 Smoking and drinking

We collected data on the personal history of smoking and drinking in 183 patients (84 males and 99 females). Smoking and drinking alcohol accounted for only 10.9% and 7.1%, respectively. However, the proportions of men who smoked and drank alcohol were 22.6% and 14.3% respectively, compared to only 1.0% and 1.0% for women, both statistically significant (P < 0.001) (**Table 1**).

### 3.2 Clinical symptoms and comorbidities

**Figure 2:**
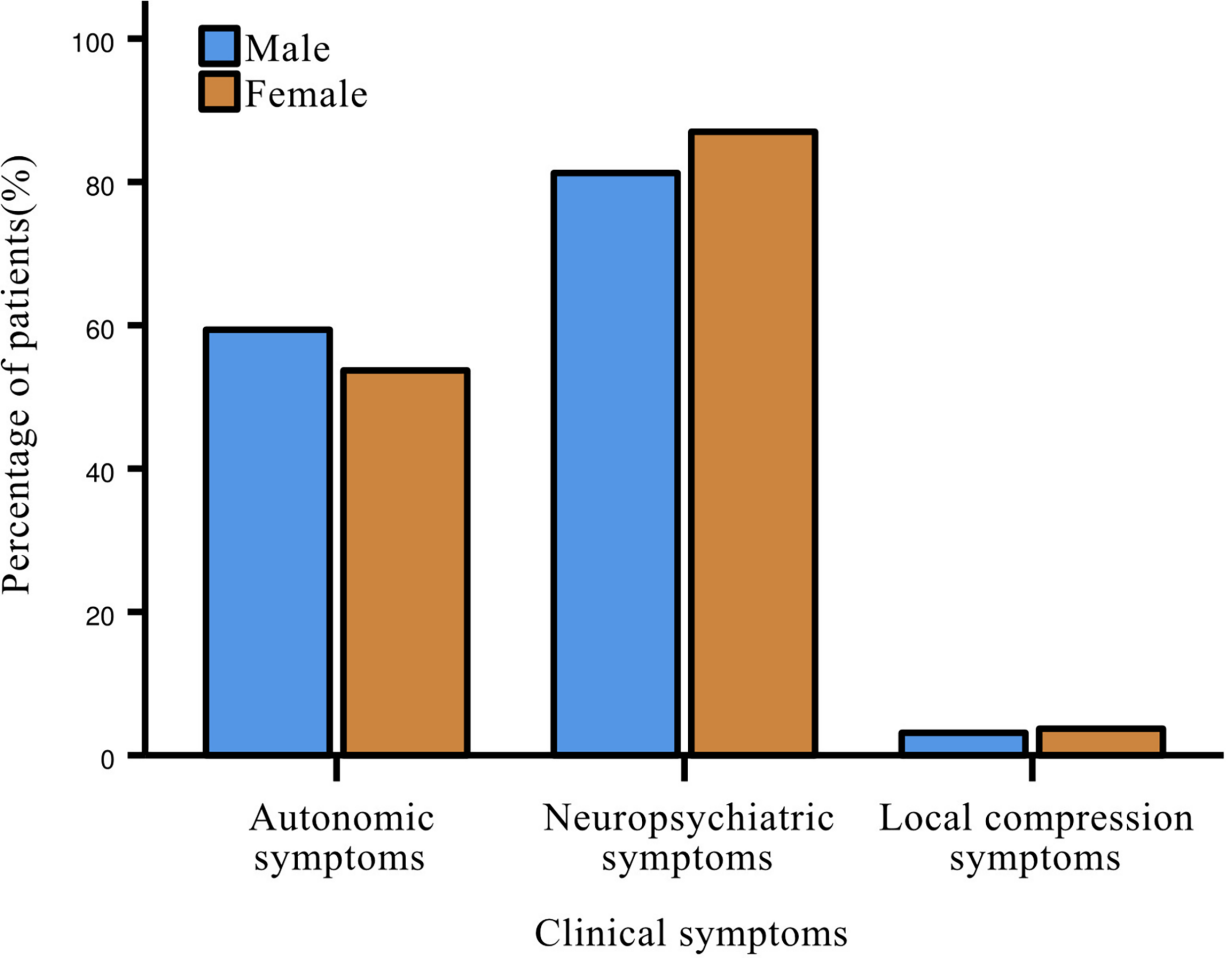
Gender differences in clinical symptoms of patients with insulinoma.

Almost all patients with insulinoma (96.5%) had clinical symptoms at disease onset, including neuropsychiatric symptoms (84.9%), most commonly dizziness; followed by autonomic symptoms (55.8%), such as tachycardia, weakness and cold sweat. Only 3.5% of patients experienced local compression symptoms (**Figure 2**). No statistical differences were present between genders in all three major categories of symptoms. Most patients with glucagonoma had symptoms of itching and rash all over the body.

**Figure 3:**
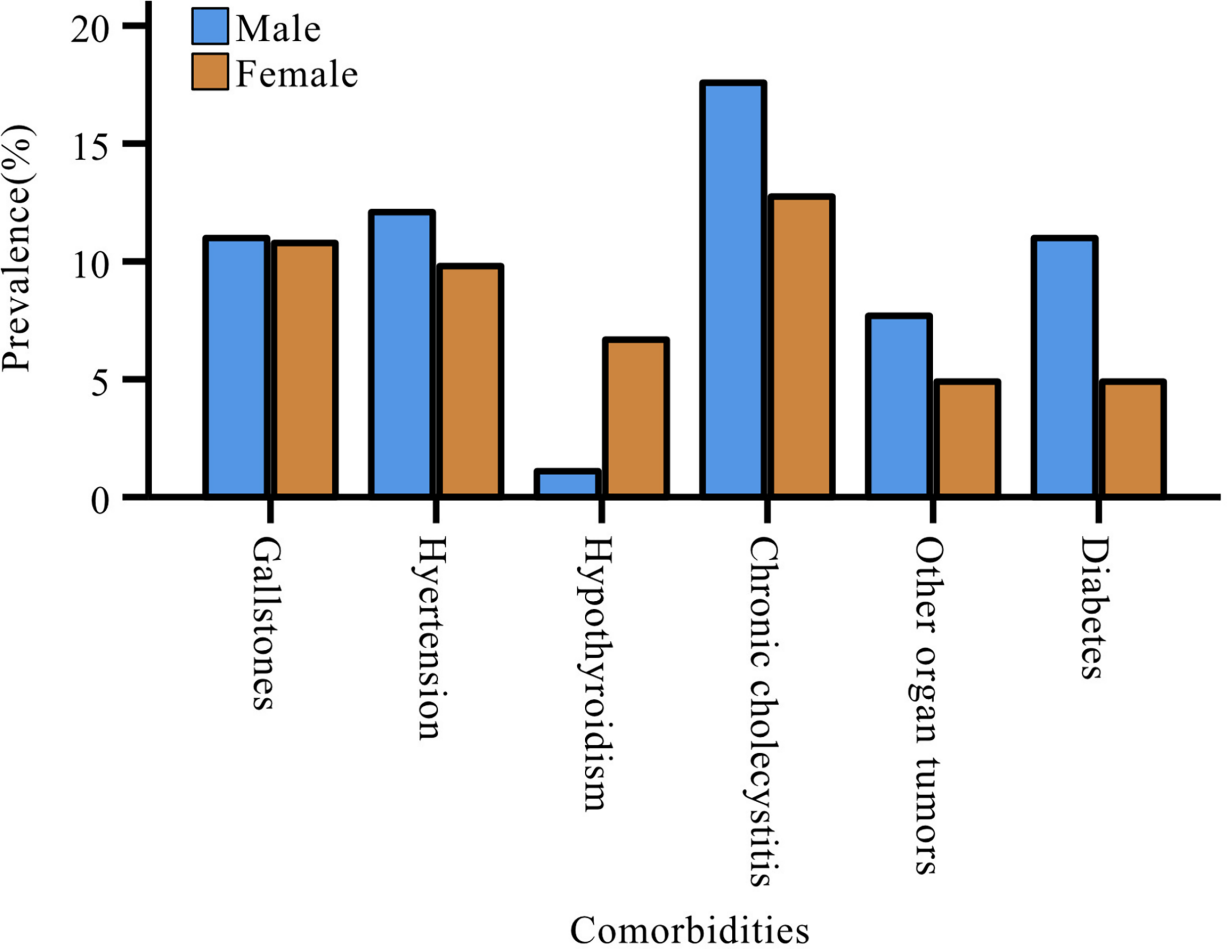
Gender differences in comorbidities of patients with pNENs.

The most common comorbidity in patients with pNENs was chronic cholecystitis (n=29, 15.0%), followed by gallstones (n=21, 10.9%) and hypertension (n=21, 10.9%). Women with pNENs had a higher rate of hypothyroidism than men (women: 6.9%; men: 1.1%), but the proportion of men with other comorbidities was more than that of women (**Figure 3**). Nevertheless, the gender differences relative to comorbidities did not reach statistical significance.

### 3.3 Characteristics of tumors

#### 3.3.1 Size, location and subtype

The mean tumor size was 2.5 ± 1.7 cm with no gender differences (males 2.5 ± 1.8 cm vs. females 2.5 ± 1.7 cm, P=0.893). The tumors were mostly located in the body or tail of pancreas in males (n=40, 53.3%), while in females they were mostly located in the head or neck of pancreas (n=41, 51.3%). The tumors were mostly non-functional (n=103, 53.4%). NF-pNENs were more frequent in males compared to females (61.5% vs. 46.1%, p=0.032) (**Table 2**). Among F-pNENs, insulinomas were the most common (n=86, 95.6%), followed by glucagonomas (n=4, 4.4%). The percentage of insulinomas was significantly higher in females than in males (52.9% vs. 35.2%), but no statistical difference was observed.

**Table 2:** Comparison of characteristics of tumors between different genders.

Parameter	All (N=193)	Male (N=91)	Female (N=102)	Detection value	P value
Size, cm	2.5 ± 1.7	2.5 ± 1.8	2.5 ± 1.7	t=-0.135	0.893
Location, n (%)	155	75	80	x^2^ = 0.325	0.568
Head or neck	76 (49.0%)	35 (46.7%)	41 (51.3%)		
Body or tail	79 (51.0%)	40 (53.3%)	39 (48.7%)		
Subtype, n (%)				x^2^ = 4.619	**0.032**
Functional	90 (46.6%)	35 (38.5%)	55 (53.9%)		
Non-functional	103 (53.4%)	56 (61.5%)	47 (46.1%)		
Grade, n (%)	167	79	88	z=-0.770	0.441
G1	92 (55.1%)	46 (58.2%)	46 (52.3%)		
G2+G3	75 (44.9%)	33 (41.8%)	42 (47.7%)		
Stage, n (%)	170	83	87	z=-1.083	0.279
I	62 (36.5%)	29 (34.9%)	33 (37.9%)		
II	85 (50.0%)	39 (47.0%)	46 (52.9%)		
III	4 (2.3%)	1 (1.2%)	3 (3.5%)		
iV	19 (11.2%)	14 (16.9%)	5 (5.7%)		

The meaning of the bold values is P<0.05.

#### 3.3.2 Grade and stage

Pathological data was available for 167 patients, and the tumor grading was as follows: low-grade G1 (n=92, 55.1%) was the most common, followed by mid-grade G2 (n=71, 42.5%), and high-grade G3 was rare (n=4, 2.4%). The proportion of males with G1 was slightly higher than that of females (58.2% vs. 52.3%), the difference was not statistically significant (P=0.441). Staging included 36.5% stage I,50.0% stage II, and 13.5% stage III and IV; there was no statistical difference between genders (P=0.279) (**Table 2**).

### 3.4 Distant metastasis

Distant metastases were identified in 11.4% of patients at the time of consultation, and the most common site was the liver (n=15, 68.2%). Although the number of males (n=13) was slightly more than that of females (n=9), there was no difference in the proportions (males:14.3%; females: 8.8%; P=0.233) (**Table 3**). The effect of functioning status on metastatic outcome in different gender patients was assessed using a stratified chi-square test; in women, functional status was significantly associated with metastatic outcome (P=0.007), whereas no such association was found in males (P=0.54). Additionally, functional tumors proved to be a protective factor for metastatic outcome compared to non-functional tumors in women (OR=0.090, 95% CI: 0.011~0.752), while the same result was not observed in males (**Table 4**).

**Table 3:** Gender differences in distant metastasis and prognosis.

Parameter	All (N=193)	Male (N=91)	Female (N=102)	Detection value	P value
Distant metastasis, n (%)	22 (11.4%)	13 (14.3%)	9 (8.8%)	x^2^ = 1.421	0.233
Progress, n (%)	37 (20.4%)	20 (23.5%)	17 (17.7%)	x^2^ = 0.939	0.332
PFS, months	52 (32.5,87.5)	52 (34.0,98.0)	54 (29.3,84.3)	z=-0.758	0.449

**Table 4:** Statistical results on the effect of functionality on metastatic outcomes in patients of different genders.

Gender	Detection value	P value	OR	95% CI
Male	x^2^ = 0.379	0.538	0.674	0.191~2.380
Female	x^2^ = 7.281	**0.007**	0.090	0.011~0.752

The meaning of the bold values is P<0.05.

Although we found that men (n=20, 23.5%) had a slightly higher rate of postoperative recurrence or metastasis compared with women (n=17, 17.7%), the difference was not statistically significant (P=0.332). Similarly, no difference in PFS was observed between genders (P=0.449) (**Table 3**).

## 4 Discussion

Gender differences in certain cancers are undeniable (4). pNENs, the second most common epithelial neoplasms of the pancreas (15), still fall into the category of rare diseases. Gender differences in susceptibility to pNENs have been reported abroad, and the impact of gender on this disease is gaining attention. To our knowledge, this retrospective study is the first based on gender differences in clinicopathological characteristics and risk of metastasis of pNENs in a Chinese population. The study included patients with pathologically confirmed pNENs and excluded non-pure pNENs, which allowed the study to provide a better dataset than other reports.

The key to gender differences is the sex chromosomes, and the study by Haupt S et al. found that gene expression from the sex chromosomes differs between males and females, which then leads to differences in tumor susceptibility (4). Scarpa A et al. performed genome-wide sequencing of 102 primary pNENs, characterized the mutations they carried and highlighted the key role of base excision repair defects due to inactivation of MUTYH, which encodes a DNA glycosylase (16). Recent research evidence suggested that mutants MEN1, DAXX, DMD, SETD2, ATRX and CREBBP were abundant in advanced pNENs (17). In addition, it was found that estrogen exposure could inhibit tumor growth in pNENs and that pNENs with good prognosis expressed higher estrogen receptor β (ERβ), which was associated with elevated expression of upregulated estrogen-induced genes (18, 19). Thus, estrogen appears to play an important role in the development of pNENs, but its specific mechanism of action still needs to be further investigated.

There is still a lack of large-scale epidemiological investigational data of pNENs in China, and the available foreign studies seem to show inconsistent results. In the American population, pNENs are more prevalent in men, which is the same in the Canadian population. In contrast, survey reports based on the Italian population demonstrate that pNENs tend to occur more frequently in women (9, 20, 21). This is an interesting phenomenon that prompts us to pay attention to gender differences in the prevalence of pNENs in different regions and between different ethnic groups. And it also suggests that environmental factors may have some influence on the occurrence of pNENs. The differences in medical resources and educational level in different regions may be related to these gender differences (22). Specifically, the educational level and overall medical resources of domestic urban areas are generally higher than those in rural areas. In addition, there are gender differences in the average BMI of Chinese residents, that is, the average BMI of males is higher than that of females (23). However, in this study, there were no differences in BMI between men and women, likely reflecting multifactorial influences such as age, region, and dietary habits.

Multiple meta-analyses have shown that smoking and drinking are risk factors for the occurrence of pNENs (24, 25). It is well accepted that for cardiovascular disease, diabetes, cancer and other diseases, smoking and drinking are influencing factors that potentially can be controlled (26). The two are closely related to the occurrence, development, and prognosis of certain tumors (27–29). The proportion of male smokers and drinkers was significantly higher, which may be related to the higher incidence of comorbidities in males. However, due to the heterogeneity of existing studies, the specific effects of tobacco and alcohol on pNENs are unclear (25). A prior study attested to a bidirectional relationship between diabetes and the development of pancreatic tumors (30). In addition, gallbladder disease has also been identified as a major risk factor for pancreatic cancer (31). However, whether there is a correlation between or an increased risk of these comorbidities and the development of pNENs, more evidence is needed.

Consistent with the results of existing studies, this study did not detect gender differences in any characteristics of the actual tumors, such as size, location, or grade (32). However, a recent study pointed out that there are gender differences in the pathological grading of tumors, with females presenting more often with low-grade tumors (33). The relationship between gender and tumor grade still needs to be further explored. The traditional view is that functionality is strongly associated with disease prognosis and survival (34, 35), but this remains to be clarified. Studies have found that functionality is an independent influencing factor when univariate analysis is performed, but when multifactorial analysis is performed, the association with disease prognosis and survival was not sustained (36, 37). Some studies have pointed out that the poor prognosis of patients with NF-pNENs may be due to atypical or no early clinical symptoms and late diagnosis (36). Gender appeared to be a risk factor affecting the prognosis of tumors, but this was also limited to univariate analysis (38). For patients with NF-pNENs, gender has been shown to be an independent influencing factor for prognosis (39). The disease may affect the quality of life of patients differently by gender, however, gender differences are grossly underestimated in clinical practice. Adequate understanding of gender-related differences is essential to improve the quality of life and prognosis of patients (40).

Our study found that NF-pNENs were more prevalent in men whereas F-pNENs were more prevalent in women, which is certainly one of our innovative findings, and the exact mechanism needs to be further explored. Hong X et al. used whole genome/whole exome sequencing (WGS/WES) technology to clarify genetic differences between F-pNENs and NF-pNENs, with the latter having copy number variant (CNV) amplification, copy neutrality and deletion, while the former lacked CNV deletion. NF-pNENs with CNV amplification and deletion are at increased risk of recurrence, suggesting a poor prognosis for the tumor (41). We found that there was no gender difference in tumor metastasis outcome, but when the functional status of tumors was included in the analysis, it was found that for women, the probability of metastasis of NF-pNENs was higher than that of F-pNENs, and non-functional tumors were a risk factor for distant metastasis, which was not shown in males. In addition, this study also found that the most common site of metastasis for pNENs, regardless of gender, was the liver, which has been confirmed by several studies (42–46). Therefore, for women diagnosed with NF-pNENs, systemic examinations to determine whether the tumor has metastasized, especially to the liver, should be emphasized, and this population requires ongoing, careful monitoring.

## 5 Conclusions

A total of 193 patients were collected in this study. The number of patients was quite large, which to a certain extent can reflect the epidemiology of the disease in the Chinese population, at least in the population of Hubei Province. We found that females have earlier onset and diagnosis than males. Clinicians should pay attention to strengthening the screening for the disease in the 40~60-year-old age group. In addition, there appear to be regional differences in the occurrence of the disease. There are gender differences in the residence and educational levels of patients with pNENs; males tend to be from urban areas and females tend to be from rural areas, and men have higher education levels than women. NF-pNENs are more prevalent in men and F-pNENs in women. Tailoring of management in women diagnosed with NF-pNENs should be particularly alert to the possibility of liver metastasis.

## 6 Limitations

1. This is a retrospective study with the usual associated limitations. 2. Although this study is based on a Chinese population, the population principally included residents of Hubei Province, China, the applicability of the results may be limited by the region. 3. In this study, F- pNENs were dominated by insulinomas, followed by the rare glucagonoma; gender differences in other types of F-pNENs could not be analyzed.

## Data availability statement

The original contributions presented in the study are included in the article/supplementary material. Further inquiries can be directed to the corresponding author.

## Ethics statement

The studies involving human participants were reviewed and approved by Union Hospital, Tongji Medical College, Huazhong University of Science and Technology (No. 0399 (2022)). Written informed consent from the participants’ legal guardian/next of kin was not required to participate in this study in accordance with the national legislation and the institutional requirements.

## Author contributions

HS conceived the study. HS, MF, and LYu developed the study design and methods. MF and LYu collected the data. MF conducted data analysis and wrote the manuscript. MF, LYu, LYa, YC, XC, and QH researched the data. MF and LYu contributed equally to the article and all authors approved the submitted version.

## Acknowledgments

The authors would like to express their gratitude to EditSprings (https://www.editsprings.cn) for the expert linguistic services provided.

## Conflict of interest

The authors declare that the research was conducted in the absence of any commercial or financial relationships that could be construed as a potential conflict of interest.

## Publisher’s note

All claims expressed in this article are solely those of the authors and do not necessarily represent those of their affiliated organizations, or those of the publisher, the editors and the reviewers. Any product that may be evaluated in this article, or claim that may be made by its manufacturer, is not guaranteed or endorsed by the publisher.

## References

[1] Edgren G Liang L Adami HO Chang ET. Enigmatic sex disparities in cancer incidence. Eur J Epidemiol (2012) 27(3):187–96. doi: 10.1007/s10654-011-9647-5 22212865

[2] Dunford A Weinstock DM Savova V Schumacher SE Cleary JP Yoda A. Tumor-suppressor genes that escape from X-inactivation contribute to cancer sex bias. Nat Genet (2017) 49(1):10–6. doi: 10.1038/ng.3726 PMC5206905 27869828

[3] Lee E Wen P. Gender and sex disparity in cancer trials. ESMO Open (2020) 5(Suppl 4):e000773. doi: 10.1136/esmoopen-2020-000773 32816862 PMC7440710

[4] Haupt S Caramia F Klein SL Rubin JB Haupt Y. Sex disparities matter in cancer development and therapy. Nat Rev Cancer (2021) 21(6):393–407. doi: 10.1038/s41568-021-00348-y 33879867 PMC8284191

[5] Man D Wu J Shen Z Zhu X. Prognosis of patients with neuroendocrine tumor: a SEER database analysis. Cancer Manag Res (2018) 10:5629–38. doi: 10.2147/CMAR.S174907 PMC6239108 30519109

[6] Gallo M Ruggeri RM Muscogiuri G Pizza G Faggiano A Colao A. Diabetes and pancreatic neuroendocrine tumours: which interplays, if any? Cancer Treat Rev (2018) 67:1–9. doi: 10.1016/j.ctrv.2018.04.013 29746922

[7] Dasari A Shen C Halperin D Zhao B Zhou S Xu Y. Trends in the incidence, prevalence, and survival outcomes in patients with neuroendocrine tumors in the united states. JAMA Oncol (2017) 3(10):1335–42. doi: 10.1001/jamaoncol.2017.0589 PMC5824320 28448665

[8] Caldarella A Crocetti E Paci E. Distribution, incidence, and prognosis in neuroendocrine tumors: A population based study from a cancer registry. Pathol Oncol Res (2011) 17(3):759–63. doi: 10.1007/s12253-011-9382-y 21476126

[9] Muscogiuri G Barrea L Feola T Gallo M Messina E Venneri MA. Pancreatic neuroendocrine neoplasms: does sex matter? Trends Endocrinol Metab (2020) 31(9):631–41. doi: 10.1016/j.tem.2020.02.010 32223919

[10] Benetatos N Hodson J Marudanayagam R Sutcliffe RP Isaac JR Ayuk J. Prognostic factors and survival after surgical resection of pancreatic neuroendocrine tumor with validation of established and modified staging systems. Hepatobil Pancreat Dis Int (2018) 17(2):169–75. doi: 10.1016/j.hbpd.2018.03.002 29576279

[11] Li G Tian ML Bing YT Tao LY Wang HY Jiang B. Clinicopathological features and prognosis factors for survival in elderly patients with pancreatic neuroendocrine tumor: A STROBE-compliant article. Med (Baltimore) (2019) 98(11):e14576. doi: 10.1097/MD.0000000000014576 PMC6426577 30882623

[12] Bocchini M Nicolini F Severi S Bongiovanni A Ibrahim T Simonetti G. Biomarkers for pancreatic neuroendocrine neoplasms (pannens) management-an updated review. Front Oncol (2020) 10:831. doi: 10.3389/fonc.2020.00831 32537434 PMC7267066

[13] Nagtegaal ID Odze RD Klimstra D Paradis V Rugge M Schirmacher P. The 2019 WHO classification of tumours of the digestive system. Histopathology (2020) 76(2):182–8. doi: 10.1111/his.13975 PMC7003895 31433515

[14] Amin MB Greene FL Edge SB Compton CC Gershenwald JE Brookland RK. The eighth edition AJCC cancer staging manual: continuing to build a bridge from a population-based to a more "personalized" approach to cancer staging. CA Cancer J Clin (2017) 67(2):93–9. doi: 10.3322/caac.21388 28094848

[15] Mpilla GB Philip PA El-Rayes B Azmi AS. Pancreatic neuroendocrine tumors: therapeutic challenges and research limitations. World J Gastroenterol (2020) 26(28):4036–54. doi: 10.3748/wjg.v26.i28.4036 PMC7403797 32821069

[16] Scarpa A Chang DK Nones K Corbo V Patch AM Bailey P. Whole-genome landscape of pancreatic neuroendocrine tumours. Nature (2017) 543(7643):65–71. doi: 10.1038/nature21063 28199314

[17] van Riet J van de Werken HJG Cuppen E Eskens F Tesselaar M van Veenendaal LM. The genomic landscape of 85 advanced neuroendocrine neoplasms reveals subtype-heterogeneity and potential therapeutic targets. Nat Commun (2021) 12(1):4612. doi: 10.1038/s41467-021-24812-3 34326338 PMC8322054

[18] Qiu W Christakis I Stewart AA Vodopivec DM Silva-Figueroa A Chen H. Is estrogen exposure a protective factor for pancreatic neuroendocrine tumours in female patients with multiple endocrine neoplasia syndrome type 1? Clin Endocrinol (Oxf) (2017) 86(6):791–7. doi: 10.1111/cen.13324 28273369

[19] Estrella JS Ma LT Milton DR Yao JC Wang H Rashid A. Expression of estrogen-induced genes and estrogen receptor β in pancreatic neuroendocrine tumors: Implications for targeted therapy. Pancreas (2014) 43(7):996–1002. doi: 10.1097/MPA.0000000000000203 25058880 PMC4628823

[20] Fraenkel M Faggiano A Valk GD. Epidemiology of neuroendocrine tumors. Front Horm Res (2015) 44:1–23. doi: 10.1159/000381970 26303701

[21] Hallet J Law CH Cukier M Saskin R Liu N Singh S. Exploring the rising incidence of neuroendocrine tumors: A population-based analysis of epidemiology, metastatic presentation, and outcomes. Cancer (2015) 121(4):589–97. doi: 10.1002/cncr.29099 25312765

[22] Das S Dasari A. Epidemiology, incidence, and prevalence of neuroendocrine neoplasms: Are there global differences? Curr Oncol Rep (2021) 23(4):43. doi: 10.1007/s11912-021-01029-7 33719003 PMC8118193

[23] Wang L Zhou B Zhao Z Yang L Zhang M Jiang Y. Body-mass index and obesity in urban and rural China: findings from consecutive nationally representative surveys during 2004-18. Lancet (2021) 398(10294):53–63. doi: 10.1016/S0140-6736(21)00798-4 34217401 PMC7617101

[24] Leoncini E Carioli G La Vecchia C Boccia S Rindi G. Risk factors for neuroendocrine neoplasms: A systematic review and meta-analysis. Ann Oncol (2016) 27(1):68–81. doi: 10.1093/annonc/mdv505 26487581

[25] Haugvik SP Hedenstrom P Korsaeth E Valente R Hayes A Siuka D. Diabetes, smoking, alcohol use, and family history of cancer as risk factors for pancreatic neuroendocrine tumors: A systematic review and meta-analysis. Neuroendocrinology (2015) 101(2):133–42. doi: 10.1159/000375164 25613442

[26] Yang Z Min Z Yu B. Reactive oxygen species and immune regulation. Int Rev Immunol (2020) 39(6):292–8. doi: 10.1080/08830185.2020.1768251 32423322

[27] Collaborators USBoD Mokdad AH Ballestros K Echko M Glenn S Olsen HE. The state of US health, 1990-2016: burden of diseases, injuries, and risk factors among US states. JAMA (2018) 319(14):1444–72. doi: 10.1001/jama.2018.0158 PMC5933332 29634829

[28] Alattas M Ross CS Henehan ER Naimi TS. Alcohol policies and alcohol-attributable cancer mortality in U.S. Atates. Chem Biol Interact (2020) 315:108885. doi: 10.1016/j.cbi.2019.108885 31678112

[29] Jiang H Livingston M Room R Chenhall R English DR. Temporal associations of alcohol and tobacco consumption with cancer mortality. JAMA Netw Open (2018) 1(3):e180713. doi: 10.1001/jamanetworkopen.2018.0713 30646024 PMC6324312

[30] Pusceddu S Buzzoni R Vernieri C Concas L Marceglia S Giacomelli L. Metformin with everolimus and octreotide in pancreatic neuroendocrine tumor patients with diabetes. Future Oncol (2016) 12(10):1251–60. doi: 10.2217/fon-2015-0077 26890290

[31] Fan Y Hu J Feng B Wang W Yao G Zhai J. Increased risk of pancreatic cancer related to gallstones and cholecystectomy: A systematic review and meta-analysis. Pancreas (2016) 45(4):503–9. doi: 10.1097/MPA.0000000000000502 26684857

[32] Muscogiuri G Altieri B Albertelli M Dotto A Modica R Barrea L. Epidemiology of pancreatic neuroendocrine neoplasms: A gender perspective. Endocrine (2020) 69(2):441–50. doi: 10.1007/s12020-020-02331-3 32468269

[33] Li WX Miao F Xu XQ Zhang J Wu ZY Chen KM. Pancreatic neuroendocrine neoplasms: CT spectral imaging in grading. Acad Radiol (2021) 28(2):208–16. doi: 10.1016/j.acra.2020.01.033 32111466

[34] Wang YH Lin Y Xue L Wang JH Chen MH Chen J. Relationship between clinical characteristics and survival of gastroenteropancreatic neuroendocrine neoplasms: A single-institution analysis (1995-2012) in south China. BMC Endocr Disord (2012) 12:30. doi: 10.1186/1472-6823-12-30 23194346 PMC3526557

[35] Han X Xu X Jin D Wang D Ji Y Lou W. Clinicopathological characteristics and prognosis-related factors of resectable pancreatic neuroendocrine tumors: A retrospective study of 104 cases in a single chinese center. Pancreas (2014) 43(4):526–31. doi: 10.1097/MPA.0000000000000065 PMC4206386 24658317

[36] Chen HY Zhou YL Chen YH Wang X Zhang H Ke NW. Functionality is not an independent prognostic factor for pancreatic neuroendocrine tumors. World J Gastroenterol (2020) 26(25):3638–49. doi: 10.3748/wjg.v26.i25.3638 PMC7366052 32742132

[37] Weiss V Dueber J Wright JP Cates J Revetta F Parikh AA. Immunohistochemical analysis of the wnt/β-catenin signaling pathway in pancreatic neuroendocrine neoplasms. World J Gastrointest Oncol (2016) 8(8):615–22. doi: 10.4251/wjgo.v8.i8.615 PMC4980652 27574554

[38] Li MX Li QY Xiao M Wan DL Chen XH Zhou L. Survival comparison between primary hepatic neuroendocrine neoplasms and primary pancreatic neuroendocrine neoplasms and the analysis on prognosis-related factors. Hepatobil Pancreat Dis Int (2019) 18(6):538–45. doi: 10.1016/j.hbpd.2019.03.009 30981633

[39] Sadula A Li G Xiu D Ye C Ren S Guo X. Clinicopathological characteristics of nonfunctional pancreatic neuroendocrine neoplasms and the effect of surgical treatment on the prognosis of patients with liver metastases: a study based on the SEER database. Comput Math Methods Med (2022) 2022:3689895. doi: 10.1155/2022/3689895 35720036 PMC9200579

[40] Ruggeri RM Altieri B Grossrubatcher E Minotta R Tarsitano MG Zamponi V. Sex differences in carcinoid syndrome: a gap to be closed. Rev Endocr Metab Disord (2022) 23(3):659–69. doi: 10.1007/s11154-022-09719-8 35292889

[41] Hong X Qiao S Li F Wang W Jiang R Wu H. Whole-genome sequencing reveals distinct genetic bases for insulinomas and non-functional pancreatic neuroendocrine tumours: Leading to a new classification system. Gut (2020) 69(5):877–87. doi: 10.1136/gutjnl-2018-317233 PMC7229893 31462556

[42] Riihimaki M Hemminki A Sundquist K Sundquist J Hemminki K. The epidemiology of metastases in neuroendocrine tumors. Int J Cancer (2016) 139(12):2679–86. doi: 10.1002/ijc.30400 27553864

[43] Maxwell JE Sherman SK O'Dorisio TM Bellizzi AM Howe JR. Liver-directed surgery of neuroendocrine metastases: what is the optimal strategy? Surgery (2016) 159(1):320–33. doi: 10.1016/j.surg.2015.05.040 PMC4688152 26454679

[44] Barat M Soyer P Al Sharhan F Terris B Oudjit A Gaujoux S. Magnetic resonance imaging may be able to identify the origin of neuroendocrine tumor liver metastases. Neuroendocrinology (2021) 111(11):1099–110. doi: 10.1159/000513015 33190136

[45] Vaghaiwalla T Keutgen XM. Surgical management of pancreatic neuroendocrine tumors. Surg Oncol Clin N Am (2020) 29(2):243–52. doi: 10.1016/j.soc.2019.11.008 32151358

[46] Cheng Y Wu D Wang L Liu H Xiong Y Xu J. Cystic pancreatic neuroendocrine tumors represent a distinct clinical entity with less aggressive biological behaviors. J Surg Res (2021) 260:134–40. doi: 10.1016/j.jss.2020.11.054 33340866

